# Cost-effectiveness of different surgical treatment approaches for early breast cancer: a retrospective matched cohort study from China

**DOI:** 10.1186/s12885-021-07840-6

**Published:** 2021-02-02

**Authors:** Qing Yang, Xiaorong Zhong, Wei Zhang, Ting Luo, Ping He, Hong Zheng

**Affiliations:** 1 Institute of Hospital Management, West China Hospital, Sichuan University, Chengdu, China; 2 Department of Head, Neck and Mammary Gland Oncology, Cancer Center, West China Hospital, Sichuan University, Chengdu, China; 3 Laboratory of Molecular Diagnosis of Cancer, Clinical Research Center for Breast, West China Hospital, Sichuan University, Chengdu, China; 4 West China Biomedical Big Data Center, West China Hospital, Sichuan University, 37 Guo Xue Alley, Chengdu, 610040 Sichuan China

**Keywords:** Breast cancer, Cost-effectiveness, Mastectomy, Breast-conserving therapy, Breast reconstruction

## Abstract

**Background:**

Both breast-conserving surgery and breast reconstruction surgery are less popular in China, although they can improve patients’ quality of life. The main reason comes from the economy. There is currently no economic evaluation of different surgical treatment options for early breast cancer. Our study aims to assess the economic impact and long-term cost-effectiveness of different surgical treatments for early breast cancer. The surgical approaches are including mastectomy (MAST), breast-conserving therapy (BCT), and mastectomy with reconstruction (MAST+RECON).

**Methods:**

Based on demographic data, disease-related information and other treatments, we applied propensity score matching (PSM) to perform 1: 1 matching among patients who underwent these three types of surgery in the tertiary academic medical center from 2011 to 2017 to obtain a balanced sample of covariates between groups. A Markov model was established. Clinical data and cost data were obtained from the medical records. Health utility values were derived from clinical investigations. Strategies were compared using an incremental cost-effectiveness ratio (ICER).

**Results:**

After PSM, there were 205 cases in each group. In the matched data set, the distribution of covariates was fully balanced. The total cost of MAST, MAST+RECON and BCT was $37,392.84, $70,556.03 and $82,330.97, respectively. The quality-adjusted life year (QALYs) were 17.11, 18.40 and 20.20, respectively. Compared with MAST, MAST+RECON and BCT have an ICER of $25,707.90/QALY and $14,543.08/QALY, respectively. The ICER of BCT vs. MAST was less than the threshold of $27,931.04. The reliability and stability of the results were confirmed by Monte Carlo simulation and sensitivity analysis.

**Conclusions:**

We believe that in the context of the limited resources in China, after comparing the three surgical approaches, BCT is the more cost-effective and preferred solution.

## Background

Breast cancer is the most common cancer in the world, ranking as the leading cancer among women and as the second leading cause of cancer death among women after lung cancer [1]. Breast cancer is also one of the most important malignancies in China. According to data from the National Cancer Registry Annual Report 2018 [2], the number of women with breast cancer in China in 2014 was approximately 279,000, with an incidence rate of 41. 82 per 100,000. The incidence rate has been increasing over the past 10 years. The increasing morbidity and mortality of breast cancer, which lead to high medical costs, has placed a huge burden on both families and society [3].

In recent years, under the background of increasing B-resolution and X-ray mammography, the early diagnosis rate of breast cancer has increased significantly [4]. Surgery is the main method of treating early breast cancer. The traditional surgical method is mastectomy. With the increasing emphasis on quality of life, breast-conserving surgery has begun to mature. A number of studies have shown that for early breast cancer, there is no statistically significant difference in disease-free survival and overall survival between the breast-conserving surgery plus radiotherapy group and the mastectomy group [5–7]. For patients undergoing mastectomy, breast reconstruction offers them the possibility to reshape their breasts [8]. However, breast-conserving therapy and breast reconstruction have increased the costs of treatment while improving the quality of life [9, 10]. Studies by Barlow et al. [11] have found that breast-conserving therapy may have higher short-term costs but lower long-term costs compared to mastectomy. Although most studies believe that breast-conserving therapy and breast reconstruction have higher costs, some studies have reached inconsistent conclusions, and evidence from China is lacking.

In China, breast-conserving surgeries account for only 6% of all breast cancer surgeries [12], and breast reconstruction only accounts for less than 10% [13]. Both breast-conserving surgery and breast reconstruction surgery are less popular in China, although they have increased in the past few decades [14]. Most stage I and II breast cancer cases still undergo modified radical surgery. Several studies have confirmed that the socioeconomic status of breast cancer patients, rather than their clinical status, is the main factor that determines the surgical treatment options for breast cancer patients [15, 16]. The trade-off between cost and quality of life benefits has become a decision that breast cancer patients must face. Cost-effectiveness analysis often uses data from clinical trials, but these patient populations may not always truly represent the patient population encountered in routine clinical practice [17]. Therefore, health economic evaluation of cancer using real-world research has become a research hotspot and trend [17, 18].

There is currently no economic evaluation of different surgical treatment options for early breast cancer. Therefore, the purpose of this study is to establish an economic model to evaluate the long-term cost- effectiveness of different surgical treatment for early breast cancer from a societal perspective. The research results can provide a basis for clinical treatment decisions and the formulation of medical insurance policies.

## Methods

### Patients and treatment options

Breast cancer patients were registered in the Breast Cancer Information Management System of West China Hospital, Sichuan University (Sichuan, China) since 1989. Their medical history, pathological diagnosis, and treatment information were prospectively collected by oncologists. Each patient was followed by outpatient visit or telephone at 3 to 4-month intervals within 2 years after diagnosis, 6-month intervals within 3 ~ 5 years, and then annually. Written informed consent was provided by all the patients. Ethical permission was granted by the Ethics Committee, West China School of Medicine/West China Hospital, Sichuan University (approval number 2017–255).

Because the baselines of the three groups were not consistent, we used R4.0.3 software to match the propensity scores. Based on the MAST + RECON group, the nearest-neighbor method was used for 1:1 matching. The rest of the statistics were performed using SPSS 25.0 software. The measurement data were analyzed by analysis of variance, the unordered counting data were tested by row × list chi-square tests, and the ordered counting data were tested by rank sum. All the tests were two-sided, and *p* < 0.05 indicated statistical significance.

### Model structure

**Fig. 1:**
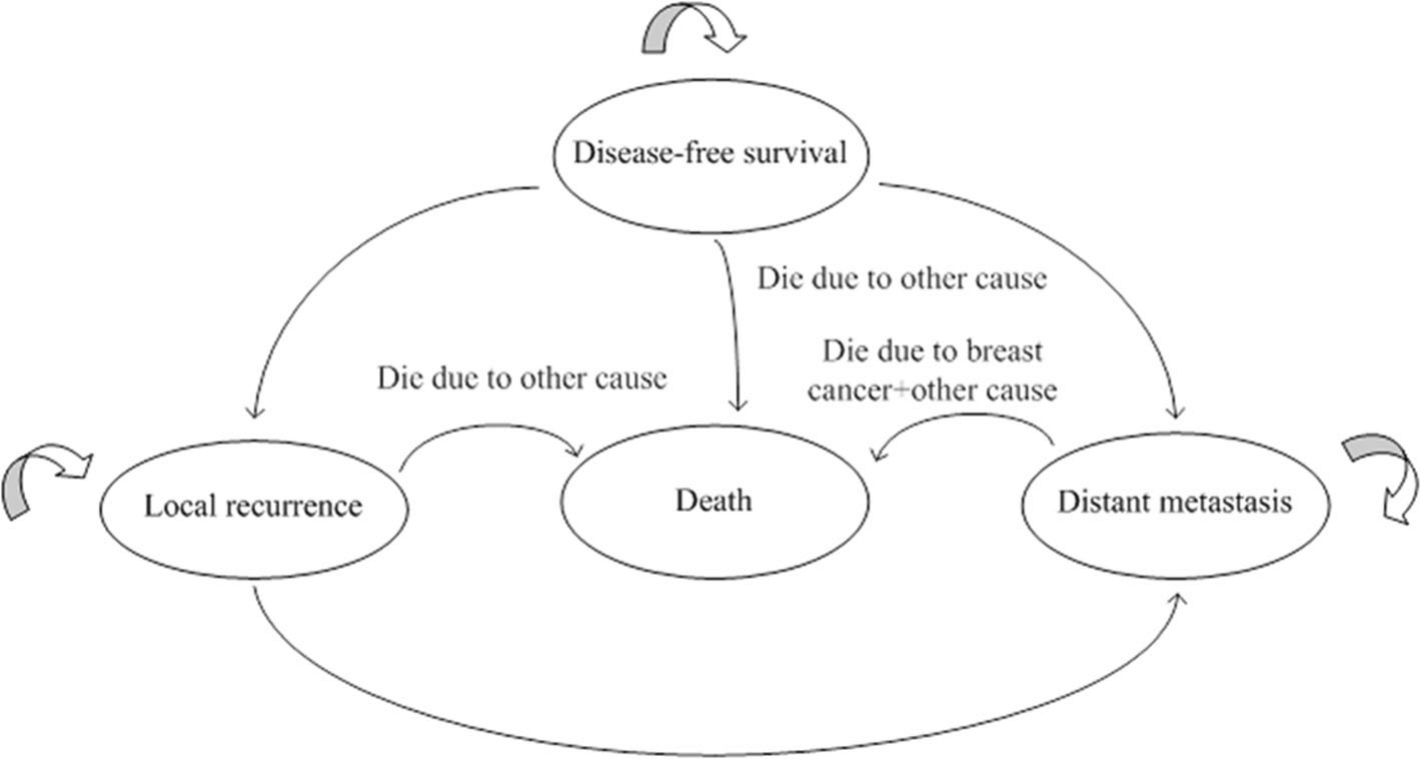
Markov basic model structure

The Markov model of early breast cancer identified in this study has four states: disease-free survival, local recurrence, distant metastasis, and death. The model was based on the following hypothesis: patients with disease-free survival can develop local recurrence and distant metastasis, patients with local recurrence can develop distant metastasis, and only patients with distant metastasis may have breast cancer-related death. It was assumed that all patients were at risk of death from causes other than breast cancer. Once a patient dies, they cannot transition to other states, so death was also an absorbed state (Fig. 1). It is assumed that the survival rate of patients can be extrapolated to the Markov model.

There are three alternative surgical options for confirmed early breast cancer patients: MAST+ RECON, BCT, and MAST. The initial age of the cohort after the propensity score in this study was 39 years. Therefore, the Markov model simulates the 60-year outcome of patients after receiving the three surgical routes. The status of all the patients entering the model was the disease-free survival status.

The utility analysis used quality-adjusted life years (QALYs), and then weighed the advantages and disadvantages of the three surgical treatment approaches. The main outcome measure used in the model was the ICER, which was the incremental cost-effectiveness ratio (ICER), that is, the ratio of the difference between the relative costs and effects of the intervention plan and those of the control plan. When comparing the ICER with the threshold, if the ICER is less than the threshold, it means that the solution is cost-effective; if the ICER is greater than the threshold, the solution is not cost-effective. The threshold for this study is willingness to pay (WTP), which uses 3 times China’s per capita gross domestic product (GDP) in 2018 [19, 20], or US $27,931.04.

TreeAge Pro 2011 (TreeAge Software, Inc., Williamstown, MA, USA) was used to build and analyze the Markov model. This software is professional software for decision trees and Markov models. This study used a 3% discount rate to discount costs and utility values and applied a half-cycle correction.

### Transition probability

In this study, the transition probability was determined by survival analysis to obtain the time to transition from one state to another state, and then the transition probability was calculated by the formula. According to the calculation formula of transition probability [21], i.e.,r = −[ln (1-P_1_)]/t_1_ and *P* = 1- ${\mathrm{e}}^{-\mathrm{r}{\mathrm{t}}_2}$, the transition probability was calculated. For example, the follow-up time from modified disease-free survival to local recurrence in this study was 94 months, with a cumulative recurrence incidence 1-P_1_ of 0.031. A Markov cycle was 12 months in a year, and the unit of follow-up time was converted from month to year to obtain the parameter t_1_. Because the annual local recurrence probability was calculated, t_2_ = 1 was taken. The local recurrence probability of MAST was calculated by the formula as 0.004019.

### Cost

This research considered the direct and indirect costs from the perspective of the whole society. All costs were expressed in US dollars ($), and the exchange rate was US $1 = 6.93 yuan (January 13, 2020). The direct cost was calculated as the direct medical costs and the patient’s transportation expenses, and the indirect cost included the patient’s lost time. Direct medical costs were derived from all inpatient and outpatient records of patients in the electronic medical record system and were collected according to the state Markov model. Since these costs came from the electronic medical record system, which included all treatment and expense records of the patient, out-of-pocket expenses were also included. The patient’s expenses include hospitalization and outpatient expenses in the following periods: the first year of treatment, the first year of recurrence, distant metastases each year, and the three months before death. Since the patient has no hospitalization expenses during the follow-up process, the follow-up expenses consist of outpatient expenses. The patient’s hospitalization costs included diagnosis, treatment, surgery, anesthesia, drugs, radiotherapy, materials, monitoring, etc. The costs for outpatients included appointments, examinations and medicine, etc. The use of resources after recurrence involved surgery, radiotherapy, chemotherapy, hormone therapy, etc., including inpatient and outpatient records.

This study also considered the first year of transportation costs for patients in different surgical treatment groups. The calculation of transportation costs was considered as the sum of the number of inpatient and outpatient visits × the average transportation cost per visit. The average transportation cost of each visit referred to the related literature published by Chengdu, China, on health economics evaluation [22]. Based on taxi fares, the transportation cost was set at 80 yuan/time.

The calculation of the cost of lost work in this study was based on the sum of the average number of days of hospitalization and the number of outpatient visits in the first year of treatment for patients in different surgical treatment groups × average daily lost time. By calculation, the loss time in the MAST + RECON group was 47 days, the loss time in the BCT group was 39 days, and the loss time in the MAST group was 44 days. According to the announcement issued by the Statistics Bureau of Sichuan Province of China, the average daily wage of employees in all units of Sichuan Province in 2018 was $9338.67/year, calculated as $25.59/day. Therefore, the lost labor cost of the MAST+ RECON group was calculated to be $1202.54, the lost labor cost of the BCT group was $940.71, and the lost labor cost of the MAST group was $1125.78.

### Health utility

It was necessary to determine the health utility value of the patients of the three surgical treatment plans within one year of treatment, after the second year or more, the cases of relapsed breast cancer within one year (state R) and those of metastatic cancer (state M). The EQ-5D-5L scale was used to investigate the health utility value of 446 Chinese breast cancer patients. The health utility value of recurrent breast cancer within one year (state R) was 0.779, and the health utility value of metastatic cancer (state M) was 0.737. The health utility values of patients undergoing BCT and MAST were also obtained from the survey. Since only 3 of the 446 patients surveyed underwent MAST + RECON, the health utilities of this surgical treatment group could not be calculated. Therefore, we used the health utility mapping model established earlier in this research group to map the value of the Functional Assessment of Cancer Therapy-Breast (FACT-B) instrument to the EuroQol-5 Dimension-5 Level (EQ-5D-5L) questionnaire to obtain the health utility of this type of patient [23]. The value of FACT-B in breast cancer patients undergoing breast reconstruction surgery was taken from the literature [24, 25], and we calculated the average value of FACT-B reported in these studies.

### Sensitivity analysis

A one-way sensitivity analysis was performed to test the robustness of the economic model and the impact of the key input parameters on the results. The results of one-way sensitivity analysis were represented by tornado diagram. The upper and lower limits of 95% confidence intervals (CI) were used as the upper and lower limits of the parameter change, and the remaining parameters adopted ±20% of the baseline value as the upper and lower limits for the parameter changes. The discount rate was set at 0 and 5% as the upper and lower limits, respectively.

For Probabilistic Sensitivity Analysis (PSA), 1000 iterations of Monte Carlo Simulation was developed to evaluate the uncertainty strategy and the results were expressed as cost-benefit acceptability curves. The distribution function was assigned to each variable of PSA to evaluate the robustness of the result. As far as the allocation for PSA is concerned, for utilities and transition probabilities, use the beta distribution, and for costs, use the lognormal distribution. The result of probability sensitivity analysis was expressed as cost- effectiveness acceptability curves.

## Results

### Patient characteristics

From 2011 to 2017, West China Hospital of Sichuan University diagnosed a total of 5070 patients with early-stage breast cancer, of which 4407 received three main types of surgery. There were 205 cases of MAST + RECON, 425 cases of BCT and 3777 cases of MAST. Taking MAST + RECON as the reference group, the propensity score matching method was used for individual matching between groups. After matching, there were 205 cases in each of the three groups. There was no significant difference in the clinical characteristics of general information, indicating that the three groups of data were balanced after matching (Table 1). We conducted survival follow-ups for the 3 groups of patients, and the deadline was April 2019.

**Table 1:** Comparison of general information after matching of propensity scores of early breast cancer patients with different surgical treatments

		MAST+ RECON (*n* = 205)	BCT (n = 205)	MAST (n = 205)	*p* value
		No.(%)	No.(%)	No.(%)	
Age,year		38.63 ±6.94	38.60 ±7.58	39.03 ±7.21	0.801
Health-care insurance	Provincial medical insurance	13 (6.34)	12 (5.85)	11 (5.37)	0.359
	City medical insurance	102 (49.76)	100 (48.78)	119 (58.05)	
	Other	90 (43.90)	93 (45.37)	75 (36.59)	
Lesion location	Left breast	110 (53.66)	112 (54.63)	109 (53.17)	0.687
	Right breast	95 (46.34)	93 (45.37)	95 (46.34)	
	Double breast	0 (0.00)	0 (0.00)	1 (0.49)	
Histology type	Ductal carcinoma in situ	10 (4.88)	5 (2.44)	6 (2.93)	0.692
	Invasive ductal carcinoma	158 (77.07)	168 (81.95)	164 (80.00)	
	Invasive lobular carcinoma	5 (2.44)	3 (1.46)	2 (0.98)	
	Other	32 (15.61)	29 (14.15)	33 (16.10)	
TNM stage	0	4 (1.95)	3 (1.46)	7 (3.41)	0.513
	I	6 (2.93)	5 (2.44)	6 (2.93)	
	II	195 (95.12)	197 (96.10)	192 (93.66)	
BMI,Kg/m2	<18.5	21 (10.24)	15 (7.32)	15 (7.32)	0.261
	18.5 ~ 23.9	144 (70.24)	164 (80.00)	148 (72.20)	
	>23.9	40 (19.51)	26 (12.68)	42 (20.49)	
Hormone receptor	Positive	105 (51.22)	106 (51.71)	195 (66.10)	0.993
	Negative	19 (9.27)	17 (8.29)	21 (7.12)	
	Mixed	73 (35.61)	75 (36.59)	73 (24.75)	
	Unkonwn / missing	8 (3.90)	7 (3.41)	6 (2.03)	
Patient source	In this city	126 (61.46)	129 (62.93)	131 (63.90)	0.899
	In this province	71 (34.63)	65 (31.71)	66 (32.20)	
	Other	8 (3.90)	11 (5.37)	8 (3.90)	
Neoadjuvant chemotherapy	No	165 (80.49)	169 (82.44)	169 (82.44)	0.840
	Yes	40 (19.51)	36 (17.56)	36 (17.56)	
Targeted therapy	No	167 (81.46)	172 (83.90)	171 (83.41)	0.786
	Yes	38 (18.54)	33 (16.10)	34 (16.59)	

Abbreviations: *MAST + RECON* Mastectomy with reconstruction; *BCT* Breast-conserving therapy; *MAST* Mastectomy

### Clinical outcome

In the current survival analysis, the Kaplan-Meier method has some certain controversies, mainly because this survival analysis method uses other competing events as censorship when estimating clinical outcome events, resulting in a higher estimate than the actual situation [26, 27]. In this study, taking into account the risk of competition, the cumulative incidence function (CIF) was used to plot the cumulative incidence of different events in each surgical group, and the significance of the difference was evaluated by Gray test. Death was treated as a competing risk event of recurrence, distant metastasis, and local recurrence to distant metastasis. Other-causes deaths were treated as competing risk events for breast cancer-specific death. The analysis was performed using packages survival,cmprsk and splines in R4.0.3 software.

**Fig. 2:**
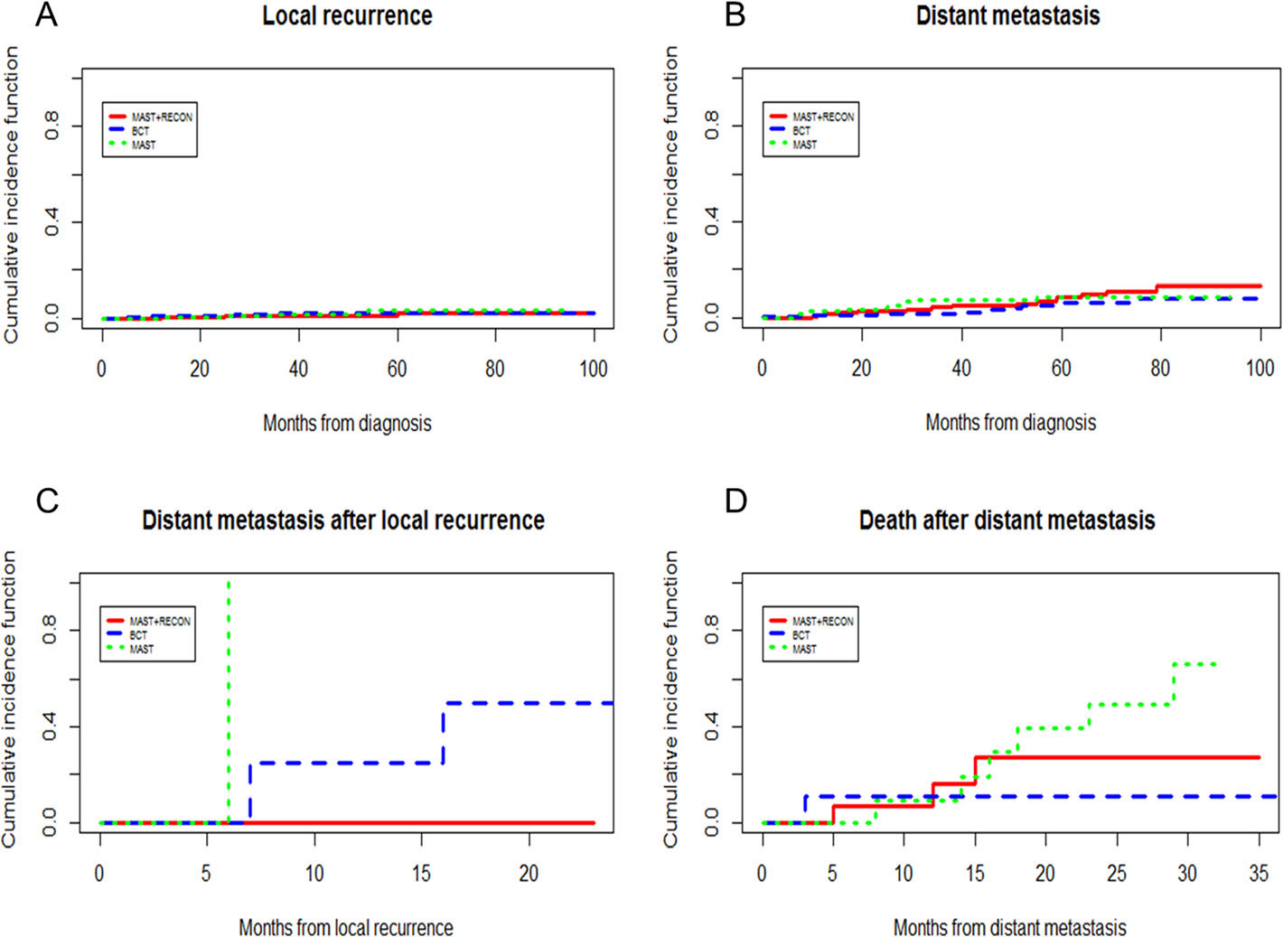
Cumulative incidence curves. **a** Cumulative incidence curve of the local recurrence. **b** Cumulative incidence curve of the distant metastasis. **c** Cumulative incidence curve of distant metastasis after local recurrence. **d** Cumulative incidence curve of death after distant metastasis

The cumulative incidence curve of each state transition is shown in Fig. 2. The cumulative incidences from disease-free survival to local recurrence and distant metastasis did not differ among the three groups (*p* = 0.892; *p* = 0.343). The cumulative incidences from local recurrence to distant metastasis were different among the three groups (*p* = 0.033). The cumulative incidences from distant metastasis to death were different among the three groups (*p* = 0.025).

### Base-case analysis

Table 2 summarizes the parameters used for model input. The results of the base-case analysis in this study are shown in Table 3. After running for 60 cycles, the total cost of the MAST group is $37,392.84, and the quality-adjusted life year is 17.11 years. The total cost of the MAST+ RECON group is $70,556.03, and the quality-adjusted life year is 18.40 years. The total cost of the BCT group is $82,330.97, and the quality-adjusted life year is 20.20 years. The strategy of using MAST leads to the lowest cost, but also the lowest quality adjustment life cycle. Compared with the MAST group, the ICERs of the MAST + RECON group and the BCT group were $25,707.90/QALY and $14,543.08/QALY, respectively. The MAST+ RECON group was rejected by extended dominance. The ICER of the BCT group was below the WTP threshold of US$27,931.04. Within this threshold, we consider that BCT is more cost-effective than the other surgical treatments and is the preferred solution.

**Table 2:** Input data values for base case, one-way sensitivity analysis and probabilistic sensitivity analysis

		Range		Distribution	Source
Parameters	Baseline	Upper boundary	Lower boundary		
Cost					
Cost of local recurrence hospitalization (first year)	10,147.18	2971.96	17,322.39	Lognormal	[a]
Cost of local recurrence outpatient (first year)	3728.43	2305.50	5151.35	Lognormal	[a]
Cost of distant metastasis hospitalization per year	10,652.42	9233.85	12,070.99	Lognormal	[a]
Cost of distant metastasis outpatient per year	2984.55	2649.14	3319.96	Lognormal	[a]
Cost of hospitalization for 3 months before death	3585.82	2533.61	4638.03	Lognormal	[a]
Cost of outpatient for 3 months before death	874.56	713.96	1035.15	Lognormal	[a]
Cost of MAST+RECON hospitalization (first year)	10,221.30	9192.74	11,249.86	Lognormal	[a]
Cost of MAST+RECON outpatient (first year)	6070.98	5233.55	6908.42	Lognormal	[a]
Cost of BCT hospitalization (first year)	7475.54	4659.50	10,291.58	Lognormal	[a]
Cost of BCT outpatient (first year)	6905.61	6051.87	7759.35	Lognormal	[a]
Cost of MAST hospitalization (first year)	6630.24	6114.63	7145.85	Lognormal	[a]
Cost of MAST outpatient (first year)	4210.88	3608.80	4812.97	Lognormal	[a]
Annual cost of follow-up for MAST+RECON	1448.12	1166.11	1730.13	Lognormal	[a]
Annual cost of follow-up for BCT	1198.82	961.66	1435.99	Lognormal	[a]
Annual cost of follow-up for MAST	770.22	770.22	1090.77	Lognormal	[a]
Transportation cost of MAST+RECON	253.97	203.17	304.76	Lognormal	[22]
Cost of losing work of MAST+RECON	1202.54	962.03	1443.04	Lognormal	[b]
Transportation cost of BCT	219.34	175.47	263.20	Lognormal	[22]
Cost of losing work of BCT	997.85	798.28	1197.42	Lognormal	[b]
Transportation cost of MAST	230.88	184.70	277.06	Lognormal	[22]
Cost of losing work of MAST	1125.78	900.62	1350.93	Lognormal	[b]
Utilities					
Local recurrence (first year)	0.779	0.641	0.917	Beta	[23]
Distant metastasis	0.737	0.657	0.817	Beta	[23]
Disease-free for MSAT+RECON (first year)	0.868	0.694	1	Beta	[24, 25]
Disease-free for MSAT+RECON (subsequent year)	0.933	0.746	1	Beta	[24, 25]
Disease-free for BCT (first year)	0.872	0.823	0.921	Beta	[23]
Disease-free for BCT (subsequent year)	0.923	0.903	0.943	Beta	[23]
Disease-free for MSAT (first year)	0.785	0.729	0.842	Beta	[23]
Disease-free for MSAT (subsequent year)	0.900	0.883	0.918	Beta	[23]
Transition probability					
Local recurrence of MAST+RECON	0.002908	0.002326	0.003489	Beta	[c]
Distant metastasis of MAST+RECON	0.016702	0.013362	0.020042	Beta	[c]
Distant metastasis after local recurrence of MAST+RECON	0.000000	0.000000	0.100000	Beta	[c]
Death after distant metastasis of MAST+RECON	0.103557	0.082845	0.124268	Beta	[c]
Local recurrence of BCT	0.002679	0.002143	0.003215	Beta	[c]
Distant metastasis of BCT	0.009889	0.007911	0.011867	Beta	[c]
Distant metastasis after local recurrence of BCT	0.201328	0.161062	0.241593	Beta	[c]
Death after distant metastasis of BCT	0.016491	0.013193	0.019789	Beta	[c]
Local recurrence of MAST	0.004019	0.003215	0.004823	Beta	[c]
Distant metastasis of MAST	0.011288	0.00903	0.013545	Beta	[c]
Distant metastasis after local recurrence of MAST	1.000000	0.800000	1.000000	Beta	[c]
Death after distant metastasis of MAST	0.335161	0.268129	0.402193	Beta	[c]
Discount rate	3%	0	5%	Constant	

Source:^a^ inpatient and outpatient records of patients in the electronic medical record system; ^b^ Calculated based on the announcement issued by the Statistics Bureau of Sichuan Province of China; ^c^ From cumulative incidence function

Abbreviations: *MAST + RECON* Mastectomy with reconstruction; *BCT* Breast-conserving therapy; *MAST* Mastectomy

**Table 3:** Base-case estimates of cost and health benefits for different surgical treatment approaches

Surgical treatment approaches	Cost($)	Incremental cost($)	QALYs	Incremental QALYs	Incremental cost per QALY (ICER, $)
MAST	37,392.84	–	17.11	–	–
MAST+RECON	70,556.03	33,163.19	18.40	1.29	25,707.90
BCT	82,330.97	44,938.13	20.20	3.09	14,543.08

Abbreviations: *MAST + RECON* Mastectomy with reconstruction; *BCT* Breast-conserving therapy; *MAST* Mastectomy; *QALYs* Quality-adjusted life years; *ICER* Incremental cost-effectiveness ratio

### Sensitivity analysis

**Fig. 3:**
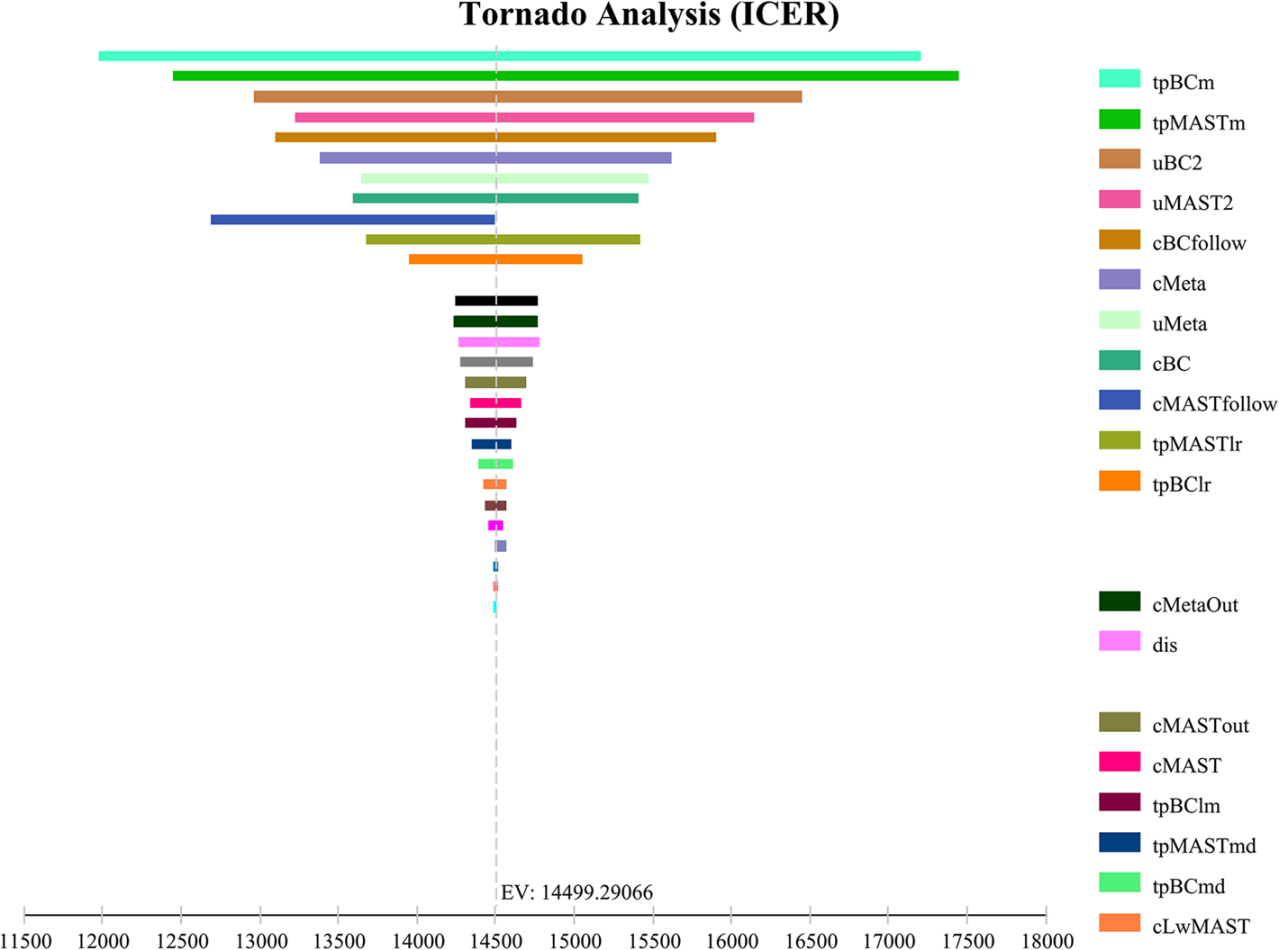
Tornado diagram presenting a one-way sensitivity analysis for BCT compared to MAST. The most influential factors are at the top of the diagram: going from the most influential to the least: tpBCm, transition probability of distant metastasis of BCT; tpMASTm, transition probability of distant metastasis of MAST; uBC2, utility of disease-free for BCT (subsequent year); uMAST2, utility of disease-free for MSAT (subsequent year); cBCfollow, annual cost of follow-up for BCT; cMeta, cost of distant metastasis hospitalization per year; uMeta, utility of distant metastasis; cBC, cost of BCT hospitalization (first year); cMASTfollow, annual cost of follow-up for MAST; tpMASTlr, transition probability of local recurrence of MAST; tpBClr, transition probability of local recurrence of BCT; cMetaOut, cost of distant metastasis outpatient per year; dis, discount rate; cMASTout, cost of MAST outpatient (first year); cMAST, cost of MAST hospitalization (first year); tpBClm, transition probability of distant metastasis after local recurrence of BCT; tpMASTmd, transition probability of death after distant metastasis of MAST; tpBCmd, death after distant metastasis of BCT; cLwMAST, cost of losting work of MAST

According to the one-way sensitivity analysis parameter variation range and the probability distribution setting of the probability sensitivity analysis listed in Table 2, we conducted a sensitivity analysis. Figure 3 shows the tornado diagrams for one-way sensitivity analysis. It can be seen from the figures that different factors have different effects on the results. The one-way sensitivity analysis of BCT and MAST showed that the three factors that have the greatest impact on ICER are the probability of disease-free survival to distant metastasis in the BCT group and the MAST group, and the health utility value of the second year in the BCT group. When all the parameters are changed within the specified range, the ICER is still lower than the WTP value.

**Fig. 4:**
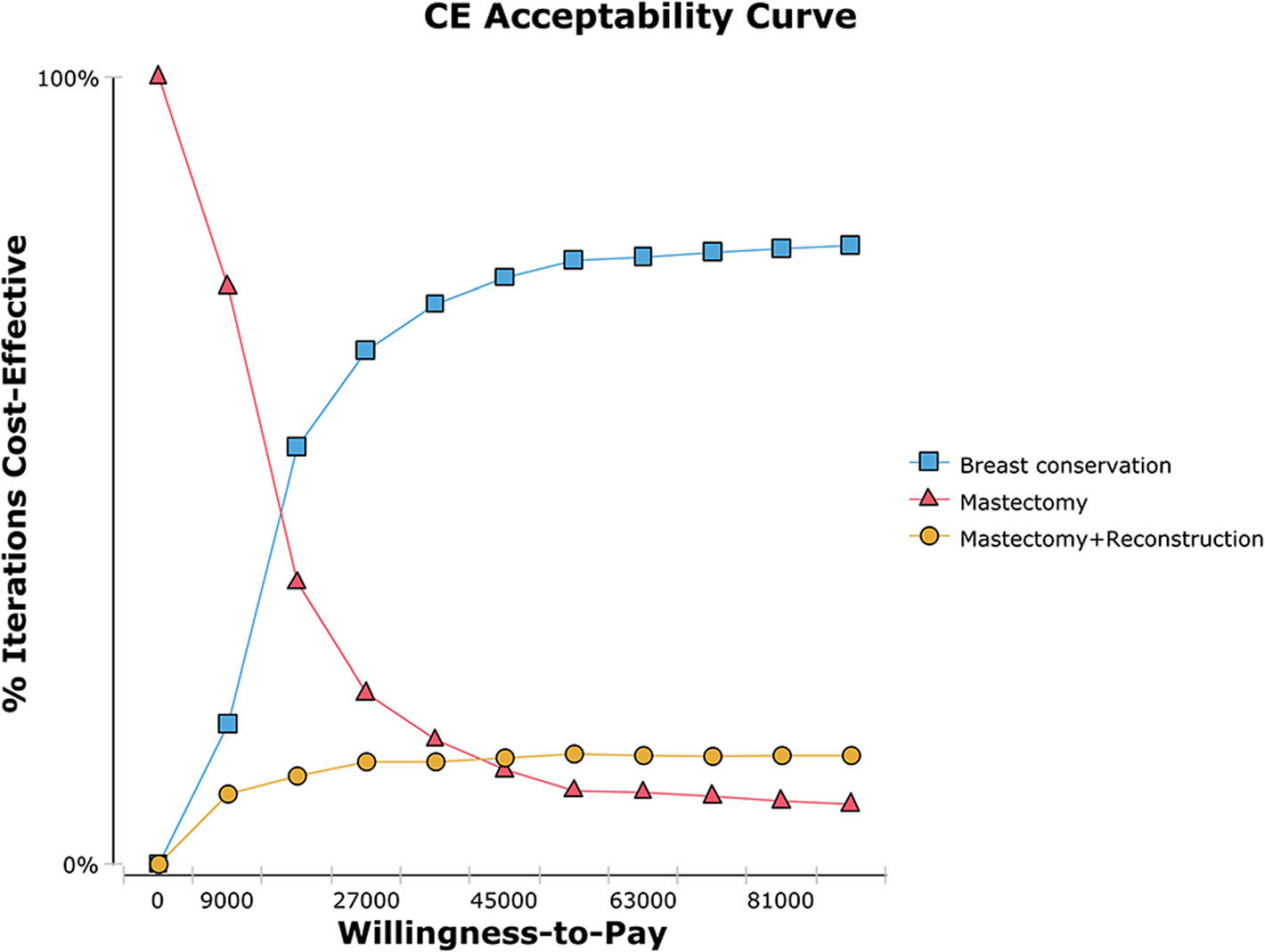
Acceptance curve of probability sensitivity analysis

**Fig. 5:**
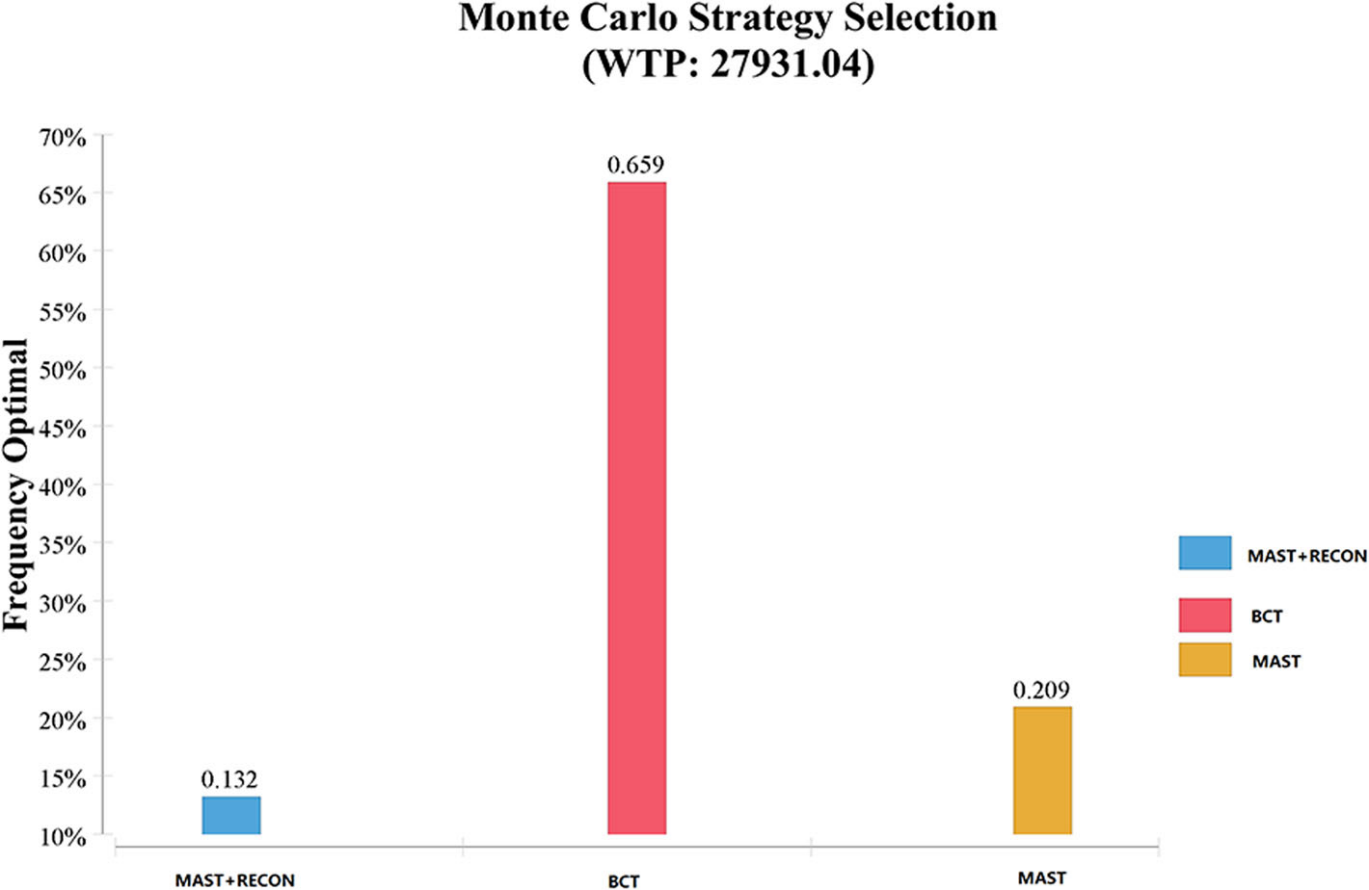
Histogram of probability sensitivity analysis

This study also conducted a probability sensitivity analysis to explore the effect of parameter distribution changes on the results. The cost parameters were lognormal distributions, and the health utility and transition probability parameters were beta distributions [28]. Figure 4 shows the acceptable curve of the probability sensitivity analysis. The results show that the probability of the cost-effectiveness of MAST decreases with an increasing WTP threshold, while that of both MAST+RECON and BCT increase with an increasing WTP threshold. When the WTP is greater than the crossing WTP value of the two acceptance curves of MAST and BCT, which is $14,543.08/QALY, the probability of the cost-effectiveness of BCT is greater than that of MAST. When the WTP is $27,931.04/QALY, the probability of choosing BCT is 65.9%, the probability of choosing a MAST is 20.9%, and the probability of choosing a MAST+RECON is 13.2% (Fig. 5).

## Discussion

Surgery is the main treatment for early breast cancer. There are many surgical options for breast cancer, including MAST, BCT, and MAST+RECON [29]. The choice of BCT and MAST+RECON is affected by the patient’s economic conditions and type of medical insurance [16], so the use ratios for these alternatives in China are low. However, compared with MAST, BCT has no significant difference in overall survivaland evidences a better quality of life [30]. Similar conclusions were obtained after modified radical mastectomy plus breast reconstruction [31]. Therefore, this study explores the cost-effectiveness of the three surgical treatment paths from the perspective of health economics to evaluate whether the increased cost-effectiveness ratio of BCT and MAST+RECON compared with MAST is cost-effective. To our knowledge, this is the first study (a) to assess the cost-utility of different surgical approaches to early breast cancer from a Chinese perspective, (b) to use real-world costs and transition probabilities, and (c) to use local cost and health utility data to build a Markov model; in addition, the health utility mapping model established in this study was used in the health utility acquisition channels. This study simulated a 60-year cohort using Markov modeling to assess the cost of obtaining each QALY. The surgical decision-making of early breast cancer patients addressed in this study can provide decision-making references for patients and medical staff and provide the basis for the formulation of relevant policies of medical insurance departments.

According to the literature published in the past, general social demographic data, disease-related conditions and patient treatment have an impact on the choice of early breast cancer surgery [16]. For example, patients with low BMI are more likely to choose modified radical mastectomy [32], while younger patients are more likely to choose breast-conserving surgery and breast reconstruction surgery [33]. Pathological staging was related to MAST, while preoperative chemotherapy was related to BCT [34]. After analyzing the cause, preoperative chemotherapy significantly increased the possibility of breast preservation [35] . In order to reduce selection bias, we performed PSM among different surgical groups based on demographic data, disease-related data, and other treatments that affect surgical treatment selection. In some published literatures, PSM was also used to match different surgical treatment groups of breast cancer, and the confounding factors considered in these literatures were also included in this study [36, 37]. After using PSM matching, the distribution of covariates among different groups is well balanced.

In the 60-year time frame, compared with MAST, the QALYs of BCT and MAST+RECON increased by 3.09 years and 1.29 years, respectively, and the cost increased by $44,938.13 and $33,163.19, respectively. Compared with the MAST group, the two groups of BCT and MAST + RECON had incremental cost-effectiveness ratios of $14,543.08/QALY and $25,707.90/QALY, respectively, of which the ICER of BCT was less than the threshold. According to this threshold, we think that the breast-conserving surgical path is more cost-effective than the other surgical treatments. When analyzing the increased QALYs of BCT compared with those of MAST, it is mainly seen that patients who undergo BCT have better a quality of life during the first year and during follow-up. In addition, the probability from distant metastasis to death in patients undergoing BCT is lower than that for the other treatment alternatives. Previous studies have found that compared with modified radical mastectomy, the difference between the local recurrence rate and distant metastasis rate is not statistically significant [5–7]. The survival analysis in this study also obtained similar conclusions. However, this study also found that patients with BCT had a lower probability of metastasis from distant metastasis to death. Studies by Fisher S et al. [38] have also showed that compared with patients receiving BCT, all-cause and breast cancer-specific death risks of patients receiving MAST are significantly higher. This outcome may cause patients with BCT to metastasize for a longer time, which will bring more costs and increase the QALYs. In a health economics evaluation study of breast cancer after breast-conserving radiotherapy conducted by Yongrui Bai et al. [39], the QALYs of patients after BCT were found to be between 8.44 and 13.79 years. The QALYs of BCT obtained in this study equal 20.20 years, which is slightly higher than the results of the previous study. The reason for the differences between the findings is that the initial age of the current study’s cohort was 39 years, which is relatively young. Because we include not only BCT, but also MAST and MAST+RECON. Many studies have shown that patients who choose BCT and MAST+RECON are younger than patients with MAST or without surgery [33, 40]. Regarding the median age of patients undergoing breast reconstruction surgery, it was 42 years old in the study of Wu ZW et al [41] The study age of Zhang L et al. is 38 years old, which is similar to the age of the subject in this study. Therefore, the average age of this study is younger than that of typical breast cancer. As more patients choose breast conserving surgery and breast reconstruction [12, 42], the findings of this study have greater guiding significance for future surgical treatments for early breast cancer.

Since most breast cancer patients are long-term survivors with high social costs, we need to accurately evaluate the cost of breast cancer compared to other malignancies [43]. Therefore, from the societal perspective, this study considers the indirect costs of breast cancer treatment in addition to the direct costs. From the perspective of indirect costs, the transportation costs and lost-time costs of breast-conserving surgery are the lowest, which may be related to less trauma regarding the operation and shorter hospital stays.

This study used one-way sensitivity analysis and probability sensitivity analysis, and the results showed that the study had stability. Below the WTP threshold, none of the parameter changes could make the MAST cost-effective.

This study has certain limitations. First, the study was based on clinical and follow-up data from confirmed patients from 2011 to 2017, with a maximum follow-up time of 9 years. Survival analysis calculates the transition probability of each state; thus, extrapolating the survival situation may not reflect the disease outcome process of patients with early breast cancer very accurately. However, a sensitivity analysis was performed in this study, which indicated that the results of this study are stable and reliable. As the patient follow-up time of the research group is extended, we will also regularly update the results of this study to make this study more accurate.

Second, the health utility value of breast reconstruction surgery did not come from our direct investigation. We found that the proportion of breast reconstruction surgery in this study was only 2%. Due to the convenience sampling used in the previous health utility survey, only 3 of 446 patients had breast reconstruction surgery, which could not meet the needs of the Markov model established in this study. Therefore, we established a mapping model of FACT-B to EQ-5D-5L [23],and mapped the FACT-B quality of life score released by China to the score of EQ-5D-5L. Although the health utility value obtained by this method is not first-hand information, when direct health utility data cannot be obtained, this method is currently the only solution for cost-utility analysis [44]. We believe that this method is more accurate than directly using the health utility value of foreign breast cancer or using the health value of other cancers. Generally, the quality of life of MAST+RECON is better than that of BCT and MAST. The health utility value of BCT obtained after mapping in this study is 0.933, which is higher than that of both MAST-RECON and MAST. The health utility value also further illustrates the effectiveness of the mapping algorithm.

## Conclusions

Overall, this study is the first to compare the different surgical treatment approaches for early breast cancer from a cost-utility perspective. The results show that, from the perspective of Chinese society, BCT is more cost-effective than both MAST and MAST + RECON. Our analysis will help clinicians make the best decisions when treating patients with early breast cancer who need surgery.

## Data Availability

The datasets used and analyzed during the current study are available from the corresponding author on reasonable request.

## Abbreviations

BCT: Breast-conserving therapy
CI: Confidence intervals
CIF: Cumulative incidence function
EQ-5D-5 L: EuroQol-5 Dimension-5 Level
GDP: Gross domestic product
FACT-B: Functional Assessment of Cancer Therapy-Breast
ICER: Incremental cost-effectiveness ratio
MAST: Mastectomy
MAST+RECON: Mastectomy with reconstruction
PSA: Probabilistic Ssensitivity Analysis
PSM: Propensity score matching
QALYs: Quality-adjusted life years
WTP: Willingness to pay

## References

[1] DeSantis CE, Ma J, Gaudet MM (2019). Breast cancer statistics,2019. CA Cancer J Clin.

[2] Wanqing C, Kexin S, Rongshou Z (2018). Cancer incidence and mortality in China, 2014. Chin J Cancer Res.

[3] Li Y, Wang K, Yin Y, Li Y, Li S (2018). Relationships between family resilience, breast cancer survivors' individual resilience, and caregiver burden: a cross-sectional study. Int J Nurs Stud.

[4] Simbrich A, Wellmann I, Heidrich J (2016). Trends in advanced breast cancer incidence rates after implementation of a mammography screening program in a German population. Cancer Epidemiol.

[5] Haffty BG, Goldberg NB, Rose M (1989). Conservative surgery with radiation therapy in clinical stage I and II breast cancer:results of a 20- year experience. Arch Surg.

[6] Blichert-Toft M, Brineker H, Andersen JA (1988). A Danish randomized trial comparing breast-preserving therapy with mastectomy in mammary carcinoma:preliminary results. Acta Oncol.

[7] Fisher B, Anderson S, Bryant J (2002). Twenty-year follow-up of a randomized trial comparing total mastectomy, lumpectomy, and lumpectomy plus irradiation for the treatment of invasive breast cancer. N Engl J Med.

[8] Lee GK, Sheckter CC (2018). Breast reconstruction following breast cancer Treatment-2018. JAMA..

[9] Sando IC, Malay S, Kozlow JH (2014). Comprehensive breast reconstruction in an academic surgical practice-an evaluation of the financial impact. Plast Reconstr Surg.

[10] Bradley CJ, Given C, Baser O (2003). Influence of surgical and treatment choices on the cost of breast cancer care. Eur J Health Econ.

[11] Barlow WE, Taplin SH, Yoshida CK (2001). Cost comparison of mastectomy versus breast-conserving therapy for early-stage breast cancer. J Natl Cancer Inst.

[12] Zhang B, Song Q, Zhang B (2013). A 10-year (1999~2008) retrospective multi-center study of breast cancer surgical management in various geographic areas of China. Breast..

[13] Huang NS, Quan CL, Ma LXX (2016). Current status of breast reconstruction in China: an experience of 951 breast reconstructions from a single institute. Gland surgery.

[14] Zhang L, Jin K, Wang X (2019). The impact of radiotherapy on reoperation rates in patients undergoing mastectomy and breast reconstruction. Ann Surg Oncol.

[15] Ali AA, Xiao H, Kiros GE (2014). Health insurance and breast-conserving surgery with radiation treatment. Am J Manag Care.

[16] Liu JJ, Zhang S, Hao X (2012). Breast-conserving therapy versus modified radical mastectomy: socioeconomic status determines who receives what--results from case-control study in Tianjin, China. Cancer Epidemiol.

[17] Petitjean A, Smith-Palmer J, Valentine W (2019). Cost-effectiveness of bevacizumab plus paclitaxel versus paclitaxel for the first-line treatment of HER2-negative metastatic breast cancer in specialist oncology centers in France. BMC Cancer.

[18] Shin S, Park CM, Kwon H (2016). Erlotinib plus gemcitabine versus gemcitabine for pancreatic cancer: real-world analysis of Korean national database. BMC Cancer.

[19] Li GN (2014). 2015 China guidelines for pharmacoeconomic evaluations and manual.

[20] Murray CJ, Evans DB, Acharya A (2000). Development of WHO guidelines on generalized cost-effectiveness analysis. Health Econ.

[21] Briggs A, Sculpher M, Claxton K (2006). Decision modelling for health economic evaluation.

[22] Huang J, Liao W, Zhou J (2018). Cost-effectiveness analysis of adjuvant treatment for resected pancreatic cancer in China based on the ESPAC-4 trial. Cancer Manag Res.

[23] Yang Q, Yu XX, Zhang W, Li H (2019). Mapping function from FACT-B to EQ-5D-5 L using multiple modelling approaches: data from breast cancer patients in China. Health Qual Life Outcomes.

[24] Jia JK, Wang Y, Guan S, Zhang KT (2014). Influences on quality of life of patients with breast cancer in different stages undergone different styles of operations. China Oncol.

[25] Wu J, Zhao Y, Wang LF (2019). Efficacy comparison of immediate breast reconstruction after breast cancer surgery and modified radical mastectomy. Cancer Res Clinic.

[26] Gooley TA, Leisenring W, Crowley J (1999). Estimation of failure probabilities in the presence of competing risks: new representations of old estimators. Stat Med.

[27] Marzona I, Baviera M, Vannini T (2016). Risk of dementia and death in patients with atrial fibrillation: a competing risk analysis of a population-based cohort. Int J Cardiol.

[28] Karnon J, Delea T, Barghout V (2008). Cost utility analysis of early adjuvant letrozole or anastrozole versus tamoxifen in postmenopausal women with early invasive breast cancer: the UK perspective. Eur J Health Econ.

[29] Chang JM, Kosiorek HE, Dueck AC (2016). Trends in mastectomy and reconstruction for breast cancer; a twelve year experience from a tertiary care center. Am J Surg.

[30] Simone NL, Dan T, Shih J (2012). Twenty-five year results of the national cancer institute randomized breast conservation trial. Breast Cancer Res Treat.

[31] Denewer A, Farouk O, Kotb S (2012). Quality of life among Egyptian women with breast cancer after sparing mastectomy and immediate autologous breast reconstruction: a comparative study. Breast Cancer Res Treat.

[32] Lucas DJ, Sabino J, Shriver CD (2015). Doing more: trends in breast Cancer surgery, 2005 to 2011. Am Surg.

[33] Kotwall C, Brinker C, Covington D (2003). Local and national trends over a decade in the surgical treatment of ductal carcinoma in situ. Am J Surg.

[34] Klemens AE, Lyndsay (2015). Factors which affect the use of lumpectomy and mastectomy in an underinsured, safety net hospital population. Am J Surg.

[35] Bonadonna G, Valagussa P, Brambilla C (1998). Primary chemotherapy in operable breast cancer: eight-year experience at the Milan cancer institute. J Clin Oncol.

[36] Sun GY, Wen G, Zhang YJ (2020). Radiotherapy plays an important role in improving the survival outcome in patients with T1-2N1M0 breast cancer - a joint analysis of 4262 real world cases from two institutions. BMC Cancer.

[37] Fan J, Wang L, Wang XJ (2006). Breast conservative therapy in east part of China: a retrospective cohort study. J Cancer Res Clin Oncol.

[38] Fisher S, Gao H, Yasui Y (2015). Survival in stage I–III breast cancer patients by surgical treatment in a publicly funded health care system. Ann Oncol.

[39] Bai Y, Ye M, Cao H (2012). Economic evaluation of radiotherapy for early breast cancer after breast-conserving surgery in a health resource-limited setting. Breast Cancer Res Treat.

[40] Morrow M, Li Y, Alderman AK (2014). Access to breast reconstruction and patient perspectives on decision making. JAMA Surg..

[41] Wu ZY, Kim HJ, Lee JW (2020). Long-term oncologic outcomes of immediate breast reconstruction vs conventional mastectomy alone for breast cancer in the setting of neoadjuvant chemotherapy. JAMA Surg.

[42] Jagsi R, Jiang J, Momoh AO (2014). Trends and variation in use of breast reconstruction in patients with breast cancer undergoing mastectomy in the United States. J Clin Oncol.

[43] Massa I, Balzi W, Burattini C (2017). The challenge of sustainability in healthcare systems: frequency and cost of inappropriate patterns of breast cancer care (the E. pic. A study). Breast..

[44] Dakin H, Abel L, Burns R (2018). Review and critical appraisal of studies mapping from quality of life or clinical measures to EQ-5D: an online database and application of the MAPS statement. Health Qual Life Outcomes.

